# Implicit opioid associations in OUD treatment: prediction of treatment response and moderation by mindfulness-oriented recovery enhancement

**DOI:** 10.1017/S0033291725102973

**Published:** 2026-01-15

**Authors:** Nina A. Cooperman, Nicole Khauli, Adam W. Hanley, Eric L. Garland

**Affiliations:** 1 Rutgers Robert Wood Johnson Medical School, USA; 2 Montefiore Medical Center, USA; 3 Florida State University, USA; 4 Department of Psychiatry, University of California San Diego School of Medicine, USA

**Keywords:** implicit association test, mindfulness, opioid use disorder, pain

## Abstract

**Background:**

Substance use is sustained partly through implicit associations toward drugs – i.e. automatic positive attitudes and motivational responses toward drug-related cues. Such implicit associations may be inferred by behavioral measures that capture the relative ease, speed, or priming of those associations. However, implicit opioid associations in patients with opioid use disorder (OUD) remain underexplored, and it is unknown whether mindfulness-based interventions such as Mindfulness-Oriented Recovery Enhancement (MORE) can modify implicit associations to support recovery.

**Methods:**

We conducted secondary analyses of data from a clinical trial of adults with OUD (N = 154), randomized to either methadone treatment as usual (TAU) or TAU plus MORE. Participants completed an opioid implicit association test (IAT) at baseline. Days of opioid use were tracked over 16 weeks. Data were analyzed using logistic and zero-inflated negative binomial (ZINB) regressions to examine the impact of baseline IAT scores on future opioid use and MORE’s moderating effect.

**Results:**

In the TAU group, each 1-unit increase in IAT D score was associated with a 216% increase in the odds of opioid use (OR = 3.16, *p* = 0.049). However, in the MORE group, IAT scores were not significantly associated with future opioid use (OR = 0.58, *p* = 0.57). ZINB analysis revealed that each 1-unit increase in IAT D score predicted 0.96 fewer days of use in MORE relative to TAU (B = –1.25; SE = 0.58; *p* = 0.030).

**Conclusions:**

Implicit attitudes toward opioids predicted higher opioid use among individuals receiving methadone. However, MORE attenuated this relationship and may counteract automatic cognitive biases that sustain opioid use.

## Introduction

Although people often initiate substance use due to conscious deliberation, addiction is thought to be maintained, in part, by implicit cognitive processes. As drug use recurs over time, conditioning processes increase the incentive salience of substance-related stimuli (Robinson & Berridge, 2000), which come to elicit compulsive and habitual drug-seeking responses coordinated by automatic drug use action schemas encoded in memory (Tiffany, 1990). Consequently, individuals with substance use disorders exhibit preferential cognitive processing of substance-related stimuli at the expense of natural, non-drug rewards. This shift in relative salience is thought to be underpinned by the dysregulatory effects of chronic drug use on hedonic homeostatic processes in brain reward circuitry (Koob, 2001). Among people with addictive behavior, positive implicit associations toward drug use relative to natural rewards may increase the risk for continued substance use.

A meta-analysis of 89 effect sizes from a range of cognitive tasks concluded that there is a significant link between implicit cognitive processes and substance use, such that individuals with more positive implicit attitudes or stronger associations toward substance use are more likely to engage in drug-using behaviors (Rooke, Hine, & Thorsteinsson, 2008). Distinct from explicit cognitive processes, implicit associations toward substances are characterized by automatic and unconscious motivational responses toward drug-related cues. Implicit associations may be inferred by behavioral measures that capture the relative ease, speed, or priming of those associations. Most addiction-related implicit association studies conducted to date have focused on implicit associations to alcohol, marijuana, and tobacco; only a few studies have evaluated implicit associations among people who use opioids. For example, Wang et al. (2015) found that implicit heroin-related cognitions were positively associated with frequency of heroin use in patients receiving methadone maintenance treatment for OUD. In a subsequent study, implicit attitudes toward heroin were positively associated with craving and the severity of heroin use disorder (Wang et al., 2021). Other research indicated that implicit associations toward opioids did not differ between temptation episodes and random assessments among individuals with OUD in a drug detoxification unit (Waters, Marhe, & Franken, 2012). However, Marhe et al. (2013) found that individuals in a similar setting who relapsed during the study reported more positive implicit attitudes toward heroin during temptation episodes but not at random assessments. Although these correlations were demonstrated cross-sectionally, it has yet to be demonstrated whether the implicit associations toward opioids can prospectively predict treatment response among people with OUD.

In addition, whether interventions can buffer the impact of implicit associations toward opioids on subsequent drug use in people in addiction recovery is not yet known. Common addiction psychotherapies (e.g. motivational interviewing, cognitive-behavioral therapy) target conscious, explicit cognitive processes (e.g. thoughts, decision making, norms, expectancies). Mindfulness-based interventions aim to increase awareness of automaticity and therefore have been proposed as a means of disrupting automatic drug use action schemas and restructuring implicit associations underpinning drug use (Garland, Froeliger, & Howard, 2014). In that regard, multiple randomized controlled trials (RCTs) have demonstrated the efficacy of mindfulness-based treatments for a range of addictive behaviors (Goldberg et al., 2018; Li, Howard, Garland, McGovern, & Lazar, 2017; Parisi, Hanley, & Garland, 2022). Ostafin et al. (2012) found that mindfulness can decouple the relation between automatic alcohol motivation and heavy drinking. This finding provides initial evidence of mindfulness-based interventions as a means of diminishing positive associations toward substance use.

Among mindfulness-based interventions, Mindfulness-Oriented Recovery Enhancement (MORE) has the strongest evidence base as a treatment for opioid misuse and OUD. Prior RCTs have demonstrated the efficacy of MORE for reducing opioid use, misuse, and craving (Cooperman, Hanley, Kline, & Garland, 2021; Garland et al., 2014, 2022, 2024). In addition, previous studies have shown that MORE can modulate implicit cognitive processes like opioid attentional bias (Garland, Baker, & Howard, 2017; Garland et al., 2024) and shift relative responsiveness to drug and natural reward stimuli (Garland, Atchley, Hanley, Zubieta, & Froeliger, 2019; Garland, Howard, Zubieta, & Froeliger, 2017). In a recent RCT of 154 people with OUD in methadone treatment, we found that adding MORE to standard addictions care significantly reduced return to drug use and decreased days of opioid use while improving methadone adherence relative to standard addictions care without MORE (Cooperman et al., 2024).

Whether MORE can buffer the impact of implicit associations toward opioids on subsequent drug use in individuals on methadone maintenance is still unknown. Therefore, we conducted a secondary analysis of data from our recent trial (Cooperman et al., 2024) where we implemented the Implicit Association Test (IAT; Greenwald et al., 1998). The IAT is one of several commonly employed measures that assess implicit (automatic) associations to emotionally salient stimuli, in addition to approach–avoidance tasks (AAT) (Wiers, Eberl, Rinck, Becker, & Lindenmeyer, 2011) as well as affective priming tasks, each of which employ distinct methods to tap into unconscious mental processes. Compared with affective priming tasks, both the IAT and AAT are generally considered more sensitive to manipulations (Znanewitz, Braun, Hensel, Altobelli, & Hattke, 2018). However, the IAT in particular is noted for exhibiting stronger reliability than many other implicit measurement approaches (Znanewitz et al., 2018). The IAT has been adapted for addiction science to study implicit associations toward substances and can assist in elucidating the cognitive processes underlying substance use behaviors and treatment outcomes. Based on existing research on implicit attitudes in OUD and preliminary studies suggesting that mindfulness might decouple implicit associations from addictive behavior (Ostafin et al., 2012), we surmised that MORE would moderate the impact of implicit opioid associations (as measured by the IAT) on addiction treatment response (i.e. future opioid use) in individuals receiving methadone. Specifically, we hypothesized that stronger implicit attitudes toward opioids at baseline would predict greater future opioid use among patients randomized to methadone treatment-as-usual (TAU). We further hypothesized that MORE – by increasing awareness of automatic associations and decoupling those associations from drug-seeking behavior – would attenuate the relationship between IAT scores and future opioid use.

## Method

### Study design and participants

This study employed a two-arm randomized controlled trial design and was approved by the Rutgers institutional review board. The primary trial was preregistered on clinicaltrials.gov (NCT04491968), but the secondary analyses in this paper were exploratory. Recruitment was conducted between August 2020 through January 2022 in five different, non-profit, methadone clinics located in urban and suburban areas of New Jersey. Participants were eligible if they were (1) 18 or older, (2) on methadone maintenance treatment for opioid use disorder, and (3) experiencing chronic pain (i.e. pain of a ≥ 3 of 10, on average, for at least 3 months) as measured by a question based on the average pain item from the Brief Pain Inventory (BPI; Cleeland & Ryan, 1994). Participants were excluded if they exhibited cognitive impairment, psychosis, or suicidal risk; were unable to attend group sessions for any reason; did not speak English; or, participated in formal mindfulness training within the past 5 years. After receiving a complete description of the study, eligible participants provided written informed consent.

### Randomization and blinding

Eligible participants were randomized in a 1:1 ratio to methadone treatment as usual (TAU) or methadone treatment plus MORE, delivered remotely, by secure phone or video conferencing. The study coordinator informed participants of their assigned condition and was the only one who had access to randomization lists, generated by computerized randomization generator. Outcome assessors, investigators, and statisticians were blinded to treatment allocation.

### Interventions

Participants randomized to the MORE arm participated in eight weekly, manualized, 2-hour group sessions delivered remotely via video conferencing. MORE sessions provided mindfulness training to engender awareness and self-regulation of automatic cognitions driving addictive behavior, reappraisal training as a means of regulating negative emotions and craving, and savoring training to increase positive emotions and augment natural reward processing. Each MORE session began with a mindfulness practice, followed by a debriefing of participants’ experiences during the practice. Psychoeducation was then provided to facilitate integration of mindfulness skills into addiction recovery and to help with pain coping. Sessions focused on using mindfulness to strengthen meta-awareness and self-regulation of automaticity, reappraisal to decrease negative affect, and savoring to increase positive affect and shift valuation from drugs back to natural healthy rewards. MORE participants were instructed to practice 15 minutes of mindfulness skills at home, guided by an audio recording. Participants in the MORE arm also received the same treatment at the methadone clinic as those randomized to methadone TAU. Methadone clinic TAU included medication management and may have included individual and group counseling sessions that did not focus on mindfulness training but were focused on motivational interviewing, cognitive-behavioral or supportive interventions, or relapse prevention as usual, at their clinic.

### Assessments

Participants completed self-report assessments, including the Addiction Severity Index, at baseline, 8 weeks, and 16 weeks, via phone, video conferencing, or in person. At the baseline visit, participants also completed a computerized IAT. Results from urine drug tests, taken during the 16-week study period, were obtained from the participants’ clinic records or participants provided a saliva sample for drug testing at baseline, 8 weeks, or 16 weeks. In addition, participants completed three daily ecological momentary assessments (EMA, prompted by randomly timed text messaging within three daily assessment windows) throughout the 16-weeks of study participation as an additional means of tracking substance use. Participants were compensated with gift cards of $30, $40, and $50 for baseline and follow-up assessments, $40 for the IAT and other cognitive tests, up to $50 for EMA assessments, and $5 for each intervention visit.

### Outcome measures

#### Days of opioid use

The Addiction Severity Index was used to assess number of days of opioid use in the past 30 days at baseline, 8 weeks, and 16 weeks. Opioid use in the past 24 hours was assessed by daily EMA prompts, delivered daily for 16 weeks. Urine drug screen results obtained during the 16-week study period were abstracted from the participants’ clinic charts, and saliva drug screens were obtained at study follow-up visits. One day of opioid use for each positive drug screen was coded for participants who did not self-report any opioid use. The greatest number of days of opioid use recorded through EMA, the ASI, or drug screen was used to calculate number of days of drug use, summed across the 16-week follow-up period.

#### Implicit opioid associations

The IAT with word-based stimuli was administered using the Inquisit software (version 6, 2021) to assess participants’ implicit associations toward opioids (Supplement). In this computerized reaction-time task, subjects respond to four categories of words using two response keys on a keyboard. The target items consisted of opioid-related words (e.g. oxycodone, heroin) adapted from prior studies using an opioid IAT (Marhe et al., 2013; Waters, Marhe, & Franken, 2012) while the contrast items consisted of natural reward words (e.g. dessert, sex). During the task, each word appears in the center of the screen, with two attribute labels – one positive (e.g. good) and one negative (e.g. bad) – displayed in the upper left and right corners. Participants are instructed to categorize each word as quickly and accurately as possible by pressing the key corresponding to the correct side of the screen. Across different blocks of trials, the pairings between items and attributes (e.g. ‘opioid + good’ vs. ‘opioid + bad’) are reversed, allowing for response time comparisons under congruent and incongruent conditions. If two concepts are more strongly associated, participants will respond faster when those concepts share the same response key. For example, individuals who have a stronger positive implicit association toward opioids than toward natural rewards will classify opioid-related words more quickly when ‘opioid’ and ‘good’ share a key than when ‘opioid’ and ‘bad’ share a key. The IAT’s updated scoring algorithm has been shown to have satisfactory internal consistency (Nosek, Greenwald, & Banaji, 2007a; Schmukle & Egloff, 2005). Studies have also supported the IAT’s convergent and discriminant validity such that IAT scores correlate moderately with other implicit measures and weakly with explicit self-report measures (Cunningham, Preacher, & Banaji, 2001; Hofmann, Gawronski, Gschwendner, Le, & Schmitt, 2005).

The IAT scoring followed the D-score algorithm proposed by Greenwald, Nosek, and Banaji (1998). The D-score reflects the difference in average response times between the critical blocks (e.g. *Opioid + Good* versus *Natural Reward + Bad* and the reverse). Faster response times in one pairing over the other indicate stronger implicit associations. Scores range from −2 to +2, where positive scores suggest a stronger implicit association between *Opioid* and *Good*, and negative scores suggest a stronger association between *Natural Reward* and *Good.* Consistent with standard IAT procedures, trials with response times below 300 ms and above 10,000 ms were excluded, and error trials were penalized by adding a 600 ms correction.

### Statistical analysis

Analyses were based on an intention-to-treat approach and were conducted using SPSS 29.0. We first conducted a logistic regression model using the SPSS PROCESS macro (Model 1) in which implicit addiction bias (IAT D score), treatment group, and the IAT D score X treatment group interaction were entered as predictors of any opioid use (measured by drug screen or self-report). We then examined IAT and treatment group effects on the number of days of opioid use through 16-week follow-up. We considered various modeling alternatives for the days of use variable (Poisson, negative binomial, zero-inflated Poisson [ZIP], zero-inflated negative binomial [ZINB]). The presence of heavy zero-inflation, in combination with overdispersion checks, and review of AIC fit statistics, indicated that ZINB was most appropriate. We thus employed a ZINB model with a logit link function to analyze days of opioid drug use through 16-week follow-up. ZINB is appropriate for modeling data with excess zeros and a high degree of overdispersion, which is common in addition research. In the ZINB model, we tested the study treatment group (MORE versus TAU) as a moderator of the association between the IAT D score and days of drug use. This model included the main effects of the treatment group, IAT D score, and the group X IAT D score interaction. We also conducted a sensitivity analysis controlling for potential confounders including methadone duration, and gender.

## Results

### Participant characteristics

Of 219 participants screened, 154 enrolled in the study, completed the IAT, and were randomized to either MORE + TAU or TAU (Table 1). Of the 154 enrolled, follow-up data on days of opioid use over 16 weeks were available for 146 participants. Details of participant enrollment and retention can be found in the primary outcomes paper (Cooperman et al., 2024). The mean age of participants was 48.5 (SD = 11.8 years) and 57% (N = 88) identified as female. Participants were diverse in race and ethnicity, with 40% identifying as Black, 13% as Hispanic, 52% as White, and 1% identified as more than one race or ethnicity. A significant portion of participants were unemployed (86%), and 23% did not have a high school diploma. At baseline, the majority of participants (53%) used opioids in the past 30 days, and participants rated their craving, on average and as measured by an adapted version of Penn Alcohol Craving Scale (PACS) (Flannery, Volpicelli, & Pettinati, 1999), as an 11.7 (SD = 8.6) on a scale from 0 to 30. Participants were taking an average methadone dose of 95 mg (SD = 40) and had been receiving methadone treatment for a mean of 3.6 years (SD = 4.6 years). Mean depression (M = 22.6, SD = 11.2) and anxiety (M = 24.4, SD = 14.7) scores were high among participants, as measured by the Centers for Epidemiological Studies Depression Scale and the Beck Anxiety Inventory (Beck, Epstein, Brown, & Steer, 1988; Radloff, 1977). There were no significant demographic differences between the MORE and the TAU groups. Seventy-three percent (n = 56) of those randomized to receive the MORE intervention completed four or more sessions (the minimum treatment dose; Cooperman et al., 2024).

**Table 1.** Sample characteristics

	Total sample (N = 154)		MORE + TAU (n = 77)		TAU (n = 77)		
Measure	N	%	n	%	n	%	Difference between groups^a^ *p*-value
Sex							
Female	88	57	44	57	44	57	1.00
Male	66	43	33	43	33	43	1.00
Race and ethnicity^b^							
Black/African American	62	40	29	38	33	43	0.511
Hispanic	20	13	11	14	9	12	0.632
White	80	52	38	49	42	55	0.519
Education, completed less than high school	36	23	19	25	17	22	0.703
Unemployed	132	86	66	86	66	86	
Primary pain condition^b^							
Back pain	35	23	21	27	14	18	0.178
Spinal conditions	34	22	21	27	13	17	0.120
Arthritis	31	20	13	17	18	23	0.315
Osteoarthritis	24	16	16	21	8	10	0.076
Other conditions (e.g. migraine, knee pain)	112	73	54	70	58	75	0.469
Used opioids in the past 30 days^c^	81	53	44	57	40	52	0.259

	M	SD	M	SD	M	SD	
Age (years)	48.5	11.8	49.2	10.9	47.7	11.3	0.405
Methadone dose (mg)	95.3	40.0	93.8	39.2	96.9	41.2	0.693
Methadone duration (years)	3.6	4.6	4.2	4.7	3.1	4.3	0.119
Depression^d^	22.6	11.2	22.5	11.6	22.6	11.0	0.105
Anxiety^e^	24.4	14.7	25.5	14.1	23.3	15.4	0.348
Baseline IAT D score^f^	−0.6	0.5	−0.6	0.5	−0.6	0.5	1.00

a Based on *X^2^* and *t* tests. No statistically significant differences existed between-groups on any variables.

b Participants could report more than one category.

c Heroin, morphine, fentanyl, or prescription pain medicine (not as prescribed).

d Measured with the Center for Epidemiological Studies Depressions scale. Higher scores suggest more depression symptoms. Scores over 16 suggest clinically significant depression (Radloff, 1977).

e Measured with the Beck Anxiety Inventory. Higher scores suggest more anxiety symptoms. Scores over 16 suggest moderate-to-severe anxiety (Beck et al., 1988).

f IAT D scores range from −2 to +2, where positive scores suggest a stronger implicit association between *Opioid* and *Good*, and negative scores suggest a stronger association between *Natural Reward* and *Good* (Nosek et al., 2007a,b; Schmukle & Egloff, 2005).

### Any opioid use

**Figure 1.**
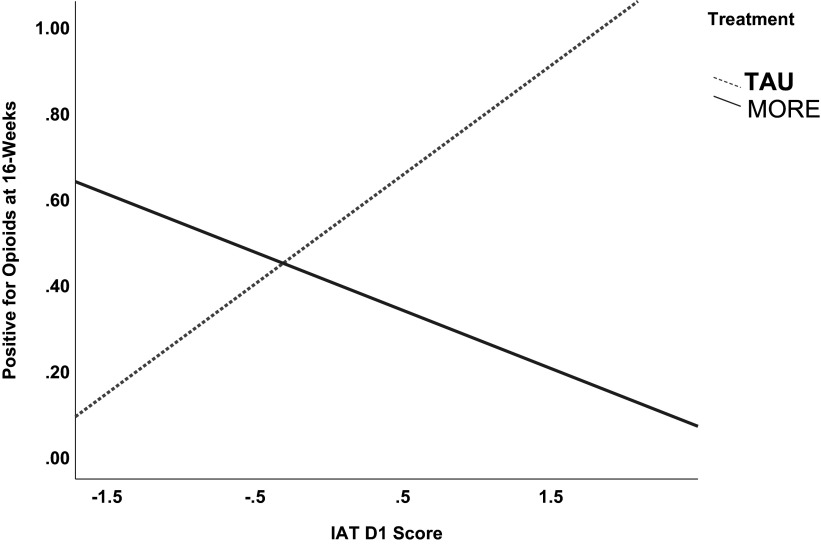
Logistic regression results indicating that treatment with MORE moderated the association between implicit associations towards opioids (D) on opioid use at 16-week follow-up (p=.028). Simple slopes analysis indicated that among the TAU group, people with higher IAT D scores were more likely to use opioids than those with lower IAT D scores (p=0.049). In contrast, among the MORE group, there was no significant association between IAD D score and future opioid use (p=0.57).

A logistic regression model revealed a significant interaction between treatment condition and implicit addiction bias (IAT D score) in predicting any opioid use (measured by drug urine screen or self-report; Figure 1). Importantly, the interaction between IAT D score and treatment group was significant (OR = 0.18; 95% CI [0.04, 0.84]; *p* = .028), indicating that the positive association between IAT scores and opioid use observed in TAU was attenuated in the MORE group. Simple slopes analysis confirmed this effect. In the TAU group, each 1-unit increase in IAT D score was associated with a 216% increase in the odds of opioid use (OR = 3.16; 95% CI [1.00, 9.97]; *p* = .049). In contrast, among participants in the MORE group, IAT scores were not significantly positively associated with future opioid use (OR = 0.58; 95% CI [0.09, 3.90]; *p* = 0.57). That is, the association between IAT D score and opioid use was approximately 5.45 times stronger in the TAU group than in the MORE group, suggesting that MORE disrupts automatic cognitive-affective mechanisms linked to future opioid use.

### Days of opioid use

A ZINB model was used to examine the interaction between treatment group (MORE versus TAU) and implicit addiction bias (IAT D score) in predicting days of opioid use. This model allowed us to simultaneously estimate the likelihood of any opioid use (zero-inflated portion) and the frequency of use among those who used (count portion), while accounting for overdispersion and excess zeros in the data (Table 2).

**Table 2.** Zero-inflated negative binomial (ZINB) model results predicting days of opioid use through 16-week follow-up (n = 146)

	Count model coefficients			
	Estimate	Std. Error	95% CI	*p*-value
Intercept	3.11	0.29	[2.54, 3.68]	<0.001
IAT D score	0.29	0.39	[−0.47, 1.05]	0.46
Treatment group	−1.17	0.46	[−2.07, −0.27]	0.01
IAT D score X treatment interaction	−1.25	0.57	[−2.37, −0.13]	0.03

	Zero-inflation model coefficients			
	Estimate	Odds ratio	95% CI	*p*-value
Intercept	−0.13	0.88	[0.48, 1.83]	0.76
IAT D score	−0.72	0.49	[0.18, 1.36]	0.17
Treatment group	0.34	1.40	[0.42, 3.03]	0.58
IAT D score X treatment interaction	1.67	5.31	[1.14, 25.17]	0.03

In the count portion of the model, the interaction between treatment condition and IAT D score was negative and significant (B = −1.25; SE = 0.58; 95% CI [−2.37, −0.13]; *p* = 0.030). MORE attenuated the association between implicit addiction biases and days of opioid use relative to TAU. Specifically, each 1-unit increase in IAT D score predicted 0.96 fewer days of use in the MORE relative to TAU. In the zero-inflated portion of the model, the interaction was positive and significant (B = 1.67; SE = 0.79; OR = 5.31; 95% CI [1.14, 25.17]; *p* = 0.034), indicating that among individuals with stronger implicit addiction biases at baseline, those in MORE group were more likely than those in the TAU group to abstain from any opioid use. That is, a 1-unit increase in IAT D score is associated with 2.58 times higher odds of being abstinent in the MORE group, compared to 0.49 times (i.e. lower odds) in the TAU. These results reflect a reversal in the direction of association due to the treatment.

## Discussion

Among OUD patients receiving methadone treatment as usual, those who had a stronger baseline implicit association toward opioid cues and away from natural rewards were significantly less likely to be abstinent over the following 16 weeks of treatment. In contrast, MORE appeared to attenuate the impact of implicit opioid associations on subsequent opioid use. That is, for patients in the MORE group, having a strong positive association toward opioids at baseline did not significantly predict later opioid use. The observed pattern suggests that MORE may not only reduce opioid use among high-risk individuals but also increase the likelihood of abstinence by shifting affective and motivational dynamics. Prior studies have demonstrated effects of MORE on implicit cognitive processes including addiction attentional bias and response inhibition (Garland et al., 2017; Garland et al., 2019; Garland, Gaylord, Boettiger, & Howard, 2010; Garland et al., 2024). To our knowledge, this is the first clinical trial to demonstrate that a mindfulness-based intervention can moderate the impact of implicit opioid associations on subsequent drug use among people with OUD.

MORE may buffer the impact of implicit opioid associations on future opioid use by disrupting automatic drug use action schema. In MORE, patients are taught to practice mindfulness in contexts where drug-related stimuli (i.e. triggers) are present as a means of increasing awareness and regulation of automatic addictive habits. When urges arise in response to opioid cues, participants are taught to practice a mindfulness of craving technique that first involves the use of meta-awareness to deconstruct the craving experience into its constituent affective, sensory, and cognitive subcomponents. Then participants are taught to engage in cognitive reappraisal by contemplating the negative consequences of satiating the craving versus the positive consequences of remaining abstinent. This dual-stage process is designed to engage top-down, conscious cognitive control over bottom-up, automatic addictive compulsions. In addition to providing training in mindfulness of craving, MORE teaches savoring techniques designed to increase responsiveness to natural healthy rewards (Garland, Froeliger, et al., 2014; Garland, Atchley, et al., 2019; Garland et al., 2023). This integration of mindfulness, reappraisal, and savoring techniques in MORE is designed to facilitate a restructuring of reward processes from valuing drug-related rewards back to valuing natural rewards, and thereby decrease addictive behavior (Garland, 2021). MORE might also buffer the impact of implicit opioid associations through this mechanism.

Neurocognitive interventions have been developed and tested as effective treatments for substance use disorders. Cognitive bias modification (CBM) (Wiers, Gladwin, Hofmann, Salemink, & Ridderinkhof, 2013) has shown to modulate implicit associations to alcohol-related stimuli (Gladwin et al., 2015) while successfully reducing neural cue reactivity and craving for alcohol use (Wiers et al., 2015) as well as relapse rates among alcohol-dependent patients (Wiers, Eberl, Rinck, Becker, & Lindenmeyer, 2011). CBM was also effective in reducing attentional bias to pain and opioid cues in individuals with OUD and chronic pain (MacLean et al., 2023). A novel theoretical framework proposes that integrating mindfulness strategies with CBM may be an optimal treatment for addictive disorders (Larsen, Hollands, Garland, Evers, & Wiers, 2023). This framework asserts that CBM and mindfulness may synergize with one another by enhancing cognitive control and weakening the link between craving and addictive responses. Given study findings that MORE may target addictive responses by weakening implicit associations to drug-related cues, optimizing MORE by combining this evidence-based behavioral therapy with CBM may yield a highly potent treatment for OUD.

Several limitations should be noted. First, the present analyses were exploratory secondary analyses of an RCT. Although the parent trial was preregistered, the moderation analyses reported here were not, and thus these findings should be interpreted with caution. Replication in future studies with pre-registered hypotheses will be important to establish the reliability and generalizability of these results. Second, although the IAT has demonstrated acceptable internal consistency and convergent validity in multiple studies, its test–retest reliability is typically modest (B. A. Nosek, Greenwald, & Banaji, 2007), which may reduce certainty about the consistency of implicit opioid associations over time. Absent a test–retest reliability analysis of the IAT, we cannot determine the stability of implicit opioid associations with future drug use or the observed moderation effects of MORE. Future studies should deploy repeated IAT measurements over the course of treatment to examine the test–retest reliability of the opioid IAT and assess whether mindfulness-based interventions produce durable modifications in implicit associations. Finally, the relatively short duration of follow-up precludes conclusions about the long-term sustainability of these effects. It remains unknown whether MORE’s moderating impact on implicit associations and opioid use is sustained in the long term. Further, in this trial, MORE was not compared to another evidence-based treatment. As such, this study cannot discern whether the observed moderating effects are unique to the MORE intervention or would also be observed for another active intervention like CBT. Future trials should examine the opioid IAT as a moderator of addiction treatment response to other evidence-based therapies.

In conclusion, treatment with MORE attenuated the influence of implicit opioid associations on subsequent opioid use, suggesting that mindfulness-based interventions can complement medication-assisted treatment by disrupting automatic, drug-related cognitive biases. These findings further suggest that measures of implicit associations can help screen and stratify risk among patients receiving standard methadone care, and that adding MORE to methadone treatment can meaningfully reduce the adverse impact of those associations on treatment outcomes.

## Supporting information

10.1017/S0033291725102973.sm001 Coooperman et al. supplementary material Coooperman et al. supplementary material

## Supplementary material

The supplementary material for this article can be found at http://doi.org/10.1017/S0033291725102973.
