# Implicit opioid associations in OUD treatment: prediction of treatment response and moderation by mindfulness-oriented recovery enhancement– ERRATUM

**DOI:** 10.1017/S0033291726103973

**Published:** 2026-03-27

**Authors:** Nina A Cooperman, Nicole Khauli, Adam W Hanley, Eric L Garland

When this article was published in Psychological Medicine it contained an error in the name of the author ‘Nina Cooperman’. This has now been corrected. The Publisher apologises for this error.
