# Supplementary material for: Implicit opioid associations in OUD treatment: prediction of treatment response and moderation by mindfulness-oriented recovery enhancement
Source: Psychol Med. 2026 Jan 15;56:e23. doi: 10.1017/S0033291725102973 (PMC12885336; doi:10.1017/S0033291725102973)
Supplement: Coooperman et al. supplementary material [file S0033291725102973sup001.docx]

**Appendix: Detailed description of the Implicit Association Test (IAT)**

**Task and Software**

The word‑based IAT was administered using Inquisit (version 6, 2021) to assess implicit associations toward opioids. The task is a computerized reaction‑time categorization that requires participants to sort stimuli into four categories using two response keys.

**Stimuli**

Target categories were opioid‑related words (e.g., oxycodone, heroin) adapted from prior opioid IAT work (Waters et al., 2012). Contrast (control) items were natural reward words (e.g., dessert, sex). Attribute categories were evaluative words labeled good and bad.

**Procedure**

Each trial displayed a single word in the center of the screen with the two attribute labels (good, bad) shown in the upper left and right corners. Participants were instructed to categorize each stimulus as quickly and accurately as possible by pressing the key corresponding to the appropriate side of the screen. The task included practice and critical test blocks in which stimulus‑to‑attribute pairings were counterbalanced and reversed across blocks (e.g., Opioid+Good vs. Opioid+Bad). Faster responding in one pairing relative to the other indicates stronger automatic association between those concepts.

**Scoring and Preprocessing**

IAT scores were computed using the D‑score algorithm described by Greenwald, Nosek, and Banaji. Preprocessing and scoring steps followed standard procedures:

Trials with response times 10,000 ms were excluded.

Error trials were retained and penalized by adding 600 ms to the recorded latency for that trial.

The D‑score was calculated as the difference in mean latencies between the two critical pairing conditions divided by the pooled standard deviation across those blocks.

D‑scores range approximately from −2 to +2, where positive values indicate a stronger implicit Opioid–Good association and negative values indicate a stronger NaturalReward–Good association.

**Psychometric Considerations**

*Reliability*: Internal split‑half and test‑retest reliabilities for single IAT administrations are typically moderate (variable across domains and stimulus sets), roughly 0.3 to 0.6 (Kvam et al., 2024). Reliability tends to improve with more trials and careful stimulus selection.

*Validity*: Convergent validity with other implicit measures is variable; prediction of behavioral, judgment, and physiological measures by the IAT had an average r ~ 0.27 (Greenwald et al., 2009).

*Sensitivity and specificity*: The IAT is not a clinical screening tool with fixed sensitivity/specificity thresholds. Its sensitivity to predict individual outcomes depends on the outcome type, context, and sample; it tends to perform better for predicting spontaneous or automatic behaviors than deliberative, self‑reported outcomes. Authors should avoid reporting single cutoff values; instead we report continuous associations and effect sizes.

**Software**: Inquisit v6 (2021).

**Block structure**: practice blocks followed by two critical test blocks with counterbalanced order across participants.

**Latency Trimming Thresholds and Error Penalty**: 10,000 ms exclusion, 600 ms error penalty.

**Predictive Scope**: The IAT captures relative, automatic evaluative associations and has been shown in many domains to predict spontaneous or automatic aspects of behavior (e.g., approach tendencies, initial liking, choice under cognitive load) and to explain incremental variance in real‑world outcomes beyond explicit measures in some settings.

**Limitations**: The IAT indexes relative associations between the specific categories included in the task (e.g., opioids vs. natural reward items) and should not be interpreted as an absolute measure of attitude magnitude. It is not a diagnostic test and does not directly measure conscious intent, detailed motives, or the full complexity of substance‑use pathology.

**References**:

Greenwald, A. G., Poehlman, T. A., Uhlmann, E. L., & Banaji, M. R. (2009). Understanding and using the Implicit Association Test: III. Meta-analysis of predictive validity. *Journal of Personality and Social Psychology*, *97*(1), 17–41. https://doi.org/10.1037/a0015575

Kvam, P. D., Irving, L. H., Sokratous, K., & Smith, C. T. (2024). Improving the reliability and validity of the IAT with a dynamic model driven by similarity. *Behavior Research Methods*, *56*(3), 2158–2193. https://doi.org/10.3758/s13428-023-02141-1

Waters, A. J., Marhe, R., & Franken, I. H. A. (2012). Attentional bias to drug cues is elevated before and during temptations to use heroin and cocaine. *Psychopharmacology*, *219*(3), 909–921. https://doi.org/10.1007/s00213-011-2424-z
